# Synergistic effects of nab-PTX and anti-PD-1 antibody combination against lung cancer by regulating the Pi3K/AKT pathway through the *Serpinc1* gene

**DOI:** 10.3389/fonc.2022.933646

**Published:** 2022-08-03

**Authors:** Jun Zhang, Zhijia Tang, Xi Guo, Yunxia Wang, Yuhong Zhou, Weimin Cai

**Affiliations:** ^1^ Department of Clinical Pharmacy, School of Pharmacy, Fudan University, Shanghai, China; ^2^ Department of Medical Oncology, Zhongshan Hospital, Fudan University, Shanghai, China

**Keywords:** albumin-bound paclitaxel, combination drug therapy, lung cancer, PD-1, *Serpinc1*

## Abstract

Lung cancer is a type of cancer with higher morbidity and mortality. In spite of the impressive response rates of nab-paclitaxel (nab-PTX) or programmed cell death-1 (PD-1) and its ligand inhibitors, the effective treatment remains limited. Currently, alternative strategies aim at drug combination of nab-PTX and PD-1/PD-L1 inhibitors. Even as the clinical impact of the combined agents continues to increase, basic research studies are still limited and the mechanisms underlying this synergy are not well studied. In this study, we evaluated the antitumor efficacy and the molecular mechanisms of action of nab-PTX in combination with anti-PD-1 antibody, using Lewis lung carcinoma (LLC) cell and subcutaneously transplanted tumor models. The combination of nab-PTX and anti-PD-1 antibody displayed stronger antitumor effects, manifested at tumor volume, proliferation and apoptosis through Ki67 and TUNEL staining. *In-vivo* experiments showed significant increases in CD4^+^ T cells, CD8^+^ T cells, IFN-γ, TNF-α, IL-2, PF, and Gzms-B, exerting antitumor effects with reductions in MDSCs and IL-10 after the treatments. Furthermore, transcriptomic analysis indicated 20 overlapped differentially expressed genes, and Serpin peptidase inhibitor clade C Member 1 (*Serpinc1*) was downregulated during treatment *in vivo*, whose expression level was markedly related to metastasis and overall survival of lung cancer patients. Functional enrichment analysis of the target gene revealed primary GO terms related to tumor, which warrants further investigation. We also found that *Serpinc1* overexpression promoted cell proliferation, migration, and invasion and inhibited cell apoptosis of LLC cells *in vitro*, possibly regulating the associated factors *via* the Pi3K/AKT pathway. In summary, our results reveal the synergistic antitumor responses of nab-PTX combined with anti-PD-1 antibody, in which *Serpinc1* may play an important role, providing a target gene for combination treatment strategy.

## Introduction

Lung cancer is the most common cancer (1) and the main cause of cancer death. The majority of lung cancer is non-small cell lung cancer (NSCLC), usually diagnosed at advanced stages (2), accounting for approximately 80% with a high metastatic potential (3). Although monotherapy with chemotherapy and immune checkpoint inhibitors (ICIs) has been widely used (4, 5), the combined agents have become a new treatment modality against NSCLC due to the resistance of chemotherapeutic drugs and limited efficacy of programmed cell death-1 (PD-1) and its ligand inhibitors alone (6, 7). Among all chemotherapeutic drugs, nanoparticle albumin-bound paclitaxel (nab-PTX) has become the preferred choice for combination therapy in clinical settings, with its unique advantages such as higher permeability, lower hematological toxicity, and no need for hormone pretreatment compared with paclitaxel (8). Moreover, in order to achieve rational drug use and develop effective drug combination regimens, it is important to understand the mechanisms behind the combined drugs, which remains unclear.

There are few basic studies on chemotherapeutic drugs combined with PD-1/PD-L1 inhibitors. A preclinical study has indicated that a combination of gemcitabine (9) and anti-PD-1 therapy modulated the tumor immune microenvironment, including tumor infiltration, in a Lewis lung carcinoma (LLC) mouse model. Other chemotherapeutic drugs, such as paclitaxel, cisplatin, or capecitabine, exerted similar effects in mouse models with different types of cancer (10, 11). Those findings provided insights into our study. However, how the combination of nab-PTX and anti-PD-1 antibody exerts a synergistic effect remains to be further clarified.

It has been reported that to improve melanoma treatment, the authors performed a transcriptomic analysis and identified a melanoblast-specific gene, *KDELR3*, the deletion of which impairs experimental metastasis (12). Transcriptomic analysis offers a powerful means for mechanistic studies and for biomarker identification, and the most common method for transcriptomic analysis is RNA sequencing, which may be an effective approach for our study. The aims of our study are to investigate whether nab-PTX plus anti-PD-1 antibody exerts synergistic effects, determine the potential therapeutic targets using mRNA sequencing data, and explore significant biological functions of the target gene. Consequently, we showed the antitumor effects of the combined agents *in vivo* and identified for the first time the important role of *Serpinc1* in lung cancer progression and treatment, including cell proliferation, apoptosis, migration, and invasion possibly *via* the Pi3K/AKT pathway.

## Materials and methods

### Cell lines, reagents, and antibodies

LLC (LL/2; LLC1) was purchased by Procell Life Science & Technology Co., Ltd. (China). Cells were cultured in DMEM medium containing 10% fetal bovine serum and 1% penicillin/streptomycin, at 37°C in an incubator with 5% CO_2_. Nab-paclitaxel injection (CSPC OUYI Pharmaceutical Co., Ltd., China) was kindly provided by Zhongshan Hospital of Fudan University (Shanghai, China). Anti-mouse PD-1 antibody (CD279) (BE0146) was purchased from Bioxcell (USA). Normal saline injection was obtained from Sinopharm Chemical Reagent Co., Ltd. (China). Primer design and synthesis were provided by Sangon Biotech Co., Ltd. (China). Mouse antibodies specific for Flag tag, Serpinc1, N-cadherin, E-cadherin, p53, Survivin, CyclinD1, Bcl-2, Bax, Pi3K, phos-Pi3K, and AKT were purchased from Proteintech (USA). Phospho-AKT (Ser473) was obtained from Cell Signaling Technology (USA). The GAPDH antibody was purchased from ABclonal (China). Mouse anti-Ly-6G/Ly-6C (Gr-1)-APC, anti-CD11b-PE, anti-CD3-PE/Cyanine7, anti-CD4-PerCP/Cyanine5.5, anti-CD8-Brilliant Violet 510™, and anti-CD45-FITC antibodies were purchased from BioLegend (USA).

### Lewis lung cancer models of C57BL/6 mice

C57BL/6 female mice (6–8 weeks old) were obtained from Shanghai Lingchang Biotechnology Co., Ltd. (China), raised in an SPF environment, free to drink and eat, and adapted to the environment for 5–7 days. Six healthy mice were assigned to a normal group without any treatment, and the others were tumor-bearing. The mouse LLC model was established with reference to the previous study (13).

### *In vivo* treatment of tumor-bearing mice

When the average tumor volume of mice reached about 50–100 mm^3^, all mice were randomly assigned to four treatment groups with different dosing schedules (**Table 1**). Mice were weighed every 3 days for the duration of the study, and the tumor volume (TV) of mice was measured and calculated every 3 days ( *TV*=*width* ^2^×*length*÷2 ). RTV means the relative tumor volume and tumor growth inhibition (TGI) value was another indicator to determine the percentage of tumor growth inhibition and antitumor efficacy *in vivo* ( *TGI*=1−*RTV*(*test*)⁄*Rtv*(*control*) ×100*%* ). At the end of the experiment, the mice were sacrificed, and their subcutaneous tumors were removed for further investigation and photographed.

**Table 1:** Experimental groups and dosing schedules of mouse LLC models (*n* = 6).

Group	Dosing schedule
Control	PBS (q3d., i.p.) + normal saline (q3d., i.v.) (d1–17)
Nab-PTX monotherapy	PBS (q3d., i.p.) + nab-PTX in normal saline (10 mg/kg, q3d., i.v.) (d1–17)
Anti-PD-1 antibody monotherapy	Anti-PD-1 antibody in PBS (200 μg, q3d., i.p.) + normal saline (q3d., i.v.) (d1–17)
Nab-PTX plus anti-PD-1 antibody	Anti-PD-1 antibody in PBS (200 μg, q3d., i.p.) + nab-PTX in normal saline (10 mg/kg, q3d., i.v.) (d1–17)

### Immunohistochemistry analysis of Ki67 and TUNEL analysis

Fresh tumor tissues after the treatment were fixed with 4% paraformaldehyde, and the steps making paraffin sections were as described previously (13). The TUNEL assay and Ki67 immunohistochemistry (IHC) analysis were performed on the sections to evaluate *in-vivo* cell proliferation and apoptosis following the manufacturer’s instructions. Slices were observed under a microscope (Nikon, Japan) and images were acquired. Cells with a brown nucleus were considered positive for Ki67, while green fluorescence staining indicated TUNEL-positive cells.

### Analysis of tumor-infiltrating lymphocytes

The single-cell suspensions of spleens and tumors were prepared as described (13). The peripheral blood and single-cell suspensions from the spleen and tumor tissues were lysed with red cell lysis buffer (BD Biosciences, USA), stained with fluorescein-conjugated antibodies (BioLegend, USA) at room temperature in the dark for 20 min, and detected on CytoFlex S flow cytometer (Beckman, USA). The following steps were processed in accordance with the manufacturer’s instructions.

### Cytokine quantification by ELISA in the tumor microenvironment

Tumor tissues were harvested as mentioned above and then homogenized cryogenically to collect the supernatants. Cytokine levels in the supernatants were measured using mouse cytokine ELISA kits (Shanghai Zhen Ke Biological Technology Co., Ltd., China) to detect the changes of IL-2, IL-10, IFN-γ, TNF-α, PF, and Gzms-B caused by nab-PTX and anti-PD-1 antibody in the tumor microenvironment according to the manufacturer’s procedures.

### RNA sequencing and bioinformatics analysis

Part of the excised tumor tissues was sequenced on an Illumina NovaSeq platform by Beijing Berry Hekang Biotechnology Co., Ltd. (China) to obtain the original sequencing data (fastq format) for biological information analysis. Comparisons of fragments per kilobase of transcript per million mapped reads level were performed using one-way ANOVA. Differential gene expression analysis for the samples between two conditions was performed using the DEGSeq R package (1.20.0). Differentially expressed genes (DEGs) were defined as those for which the adjusted *p*-value was below 0.05 and the log_2_ (fold change) was more than 1. Venn diagrams were generated using the “Venn Diagram” packages in R software, and volcano plots of differential expression data were plotted using the −log_10_ (*p*-value) and log_2_ (fold change) using the R package ggplot2. The overlapped differentially expressed genes of these three comparisons were hierarchical clustered with MeV (MultiExperiment Viewer, v4.9.0) software using Euclidian distance as the similarity metric (centered) and centroid linkage as the clustering method. Gene Ontology (GO) analysis of differentially expressed genes was implemented in the GOseq R package. GO terms with adjusted *p*-value below 0.05 were considered as significantly enriched by differential expressed genes. Moreover, the bar graphs of GO functional enrichment terms were generated using GraphPad Prism 8.

### Quantitative RT-PCR

Total RNA was extracted from tumor tissues or cells with RNAiso Plus (Takara Bio Inc., Shiga, Japan). The first-strand cDNA was generated using the HiScript III RT SuperMix for qPCR with gDNA wiper (Vazyme Biotech Co., Ltd., China), and quantitative PCR was performed according to the manufacturer’s instructions. The sequences of the primers are shown in **Table S1**. The mRNA levels of the genes were normalized to GAPDH mRNA expression.

### Correlation analysis from public databases

TNMplot (14) (https://tnmplot.com/analysis/) was used to compare *Serpinc1* gene expression level in normal (*n* = 391), tumor (*n* = 1,865), and metastatic (*n* = 8) lung tissues using gene chip data. Kaplan–Meier curves were generated using an online tool (http://kmplot.com/analysis/) (15) to investigate the association of *Serpinc1* (Affy ID: 210049_at) expression level and overall survival (OS) of lung cancer patients (*n* = 1,925). The KM plots, hazard ratio (HR) with 95% confidence intervals, and log-rank *p*-value were calculated and plotted in R software. Lung adenocarcinoma patients (*n* = 719) and squamous cell carcinoma patients (*n* = 524) were further selected to investigate this association.

### Plasmids and transfection

Mouse *Serpinc1* overexpression plasmid was constructed on the control vector (pcDNA3.1-3xFlag-C) by Hunan Fenghui Biotechnology Co., Ltd. (China). LLC cells were transfected with the control vector or *Serpinc1* overexpression plasmids using Lipofectamine 3000 (Invitrogen, USA) following the manufacturer’s instructions. The sequences for *Serpinc1* overexpression used in the present study were as follows: forward, GGCTGCTGGTGAGAGGAAG, and reverse, GGATTCACGGGGATGTCTCG. After transfection, cells were taken for qRT-PCR or Western blotting.

### Western blotting

Cells were lysed with phosphatase and protease inhibitor in RIPA lysis buffer and centrifuged at 12,000 rpm for 5 min to collect the supernatants of protein. The total protein concentration was determined by BCA assay using the BCA Assay Kit (Beyotime Biotechnology, China). Electrophoresis and membrane transfer were performed as described (16). Protein bands were detected by enhanced chemiluminescence (ECL) kit (Dalian Meilun Biotechnology Co., Ltd., China), developed by a chemiluminescence imaging system and quantified using ImageJ software (National Institutes of Health, Bethesda, MD, USA).

### Analysis of cell viability and apoptosis

For cell proliferation capacity of transfected cells, cell viability was detected at 24, 48, and 72 h post-transfection with the CCK8 kit (Dalian Meilun Biotechnology Co., Ltd., China). Briefly, cells were plated into 96-well plates containing a culture medium. Subsequently, the cells were exposed to different transfected vectors at indicated times. The CCK8 assay was carried out by adding 10 µl of CCK8 reagent into each well, incubating at 37°C for another 1 h, and cell viability was measured by testing the absorbance at 450 nm using a microplate reader. Colony formation assay was performed (17) to detect long-term cell survival with the transfection. The extent of cell apoptosis was determined using Annexin V-FITC/PI kit (Dalian Meilun Biotechnology Co., Ltd., China) according to the manufacturer’s instructions.

### Wound healing assays

Wound healing assays were conducted to evaluate the cell mobility of *Serpinc1* overexpressing LLC cells. Briefly, cells were seeded in six-well plates, and then scratches were made with a 200-μl pipette tip to wound at a cell confluence of 90%. Each well was washed twice with PBS gently and replaced with basal DMEM containing the control vector or *Serpinc1* overexpression plasmids. Cells in each well were visualized and photographed under an inverted microscope at the indicated time after wounding. The width of the scratch gap was measured using ImageJ software (National Institutes of Health, Bethesda, MD, USA). Wound healing rate was calculated as follows: *Wound* *healing* *rate*=((*initial* *wound* *width*−*wound* *width* *at* *tested* *time* *point* ))⁄(*initial* *wound* *width*)×100*%* .

### Transwell assays

Cell migration and invasion were determined by Transwell assays. Cells were transfected with the control vector or *Serpinc1* overexpression plasmids for 24, 48, and 72 h. Then after digestion, cells were resuspended, counted, and seeded in a 100-μl serum-free medium in the Transwell chamber (8 μm pore size, Corning Inc., USA), and the lower chamber was filled with 600 μl of medium containing 10% serum. After 12–24 h, the translocated cells were fixed and stained, and then images were captured by an inverted microscope. For the cell invasion assay, the upper sides of the chambers were coated with 2% Matrigel (BD Biosciences, USA) diluted in 100 μl of serum-free medium in advance. The other procedures were the same as described above.

### Statistics

Data are presented as mean ± standard deviation from at least three independent experiments. Statistical analysis was performed using GraphPad Prism 8 by two-tailed unpaired Student’s *t*-tests between two groups. One- or two-way analysis of variance (ANOVA) was used for multiple comparisons among three or more groups. Statistically significant differences are annotated with a line segment and an asterisk. Sample size, the number of replicates, and the statistical test are described in the figure captions. A *p*-value less than 0.05 is considered statistically significant.

## Results

### nab-PTX plus anti-PD-1 antibody inhibits synergistically LLC tumor growth *in vivo*

**Figure 1:**
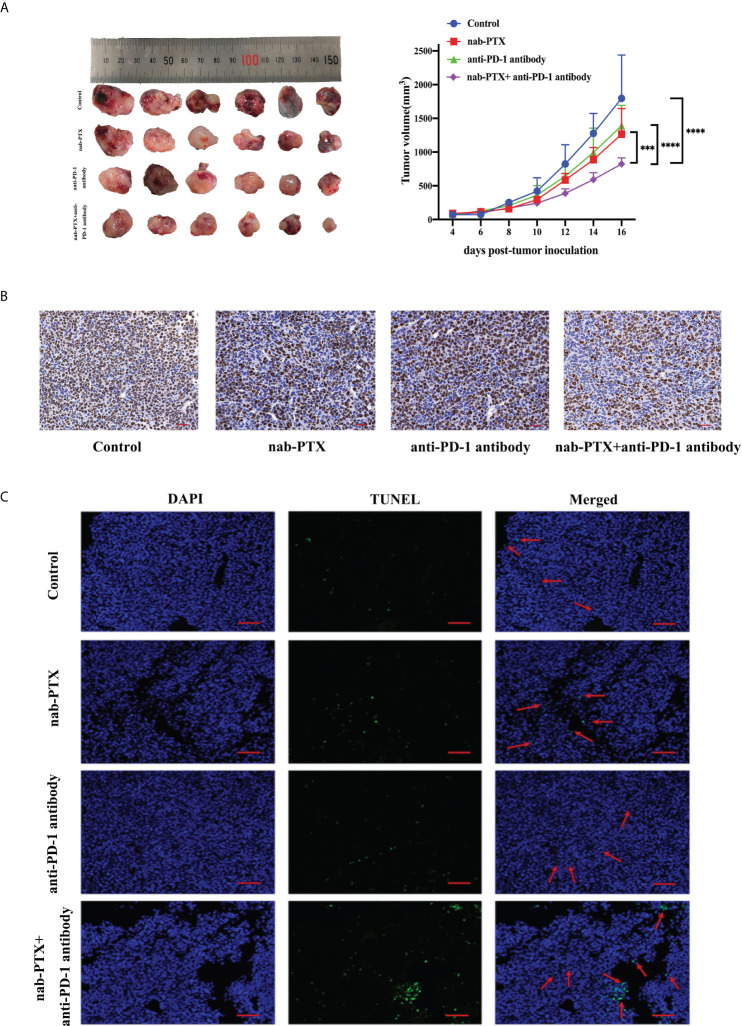
The antitumor responses of mice treated with nab-paclitaxel (nab-PTX) and anti-PD-1 antibody. **(A)** Macroscopic images and tumor growth curve in mouse LLC cancer models (*n* = 6, biological duplicates), statistically analyzed with two-way ANOVA followed by Tukey’s multiple comparisons. ***p< 0.001, ****p< 0.0001. **(B)** Immunohistochemistry staining of Ki67 for tumor sections (*n* = 3, magnification ×20, biological duplicates). The hematoxylin-stained nucleus is blue, and the positive expression of DAB is brownish yellow. **(C)** Apoptosis detection of tumor sections by TUNEL staining after the last administration (*n* = 3, magnification ×200, biological duplicates). Green, TUNEL staining; blue, DAPI. The red arrow denotes a representative TUNEL-positive cell.

To investigate the antitumor responses after a combination of nab-PTX and anti-PD-1 antibody, a subcutaneous LLC mouse model was established, and then the mice were administered different drugs. The indicated doses of both drugs were shown to be well tolerated as evaluated by monitoring the changes of murine body weight (**Figure S1**). We also found that tumors in each group grew gradually (F**igure 1A**). Monotherapy with anti-PD-1 antibody showed limited activity (TGI 29%), and nab-PTX slightly increased tumor growth inhibition (TGI 43%), while the combination treatment of nab-PTX and anti-PD-1 antibody resulted in substantially improved efficacy (TGI 60%, *p*< 0.05) compared with any monotherapy group. In addition to the indicator of tumor volume, Ki67 staining revealed a significant decrease in proliferating cancer cells with the combined agents (**Figure 1B**). Analogous to the results in Ki67 analysis, TUNEL-positive staining was observed in more areas of the tumor sections after the combination therapy of nab-PTX and anti-PD-1 antibody, suggesting a strong pro-death effect of the combination treatment *in vivo* (**Figure 1C**). These results demonstrated that the combination treatment significantly improved antitumor efficacy compared with nab-PTX or anti-PD-1 antibody alone, showing synergies *in vivo*.

### Combination treatment alters immune infiltration *in vivo*

**Figure 2:**
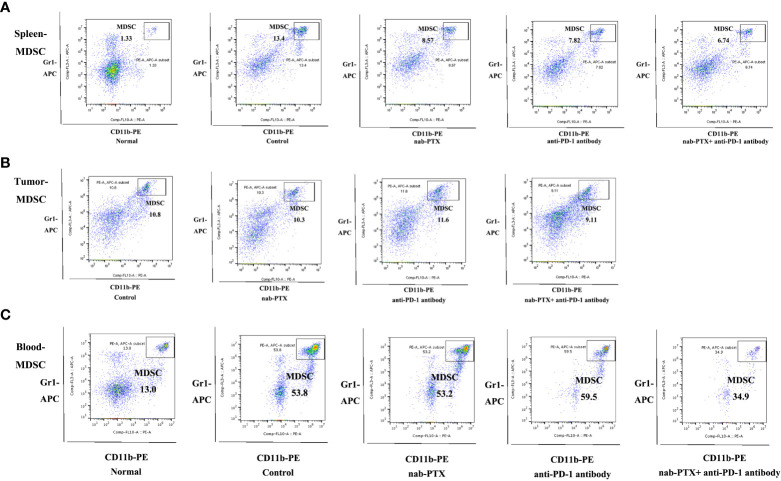
Representative flow cytometric plots of MDSCs in **(A)** spleen (*n* = 3, biological duplicates), **(B)** tumor (*n* = 5, biological duplicates), and **(C)** peripheral blood (*n* = 3, biological duplicates) after treatment with nab-PTX and anti-PD-1 antibody.

**Figure 3:**
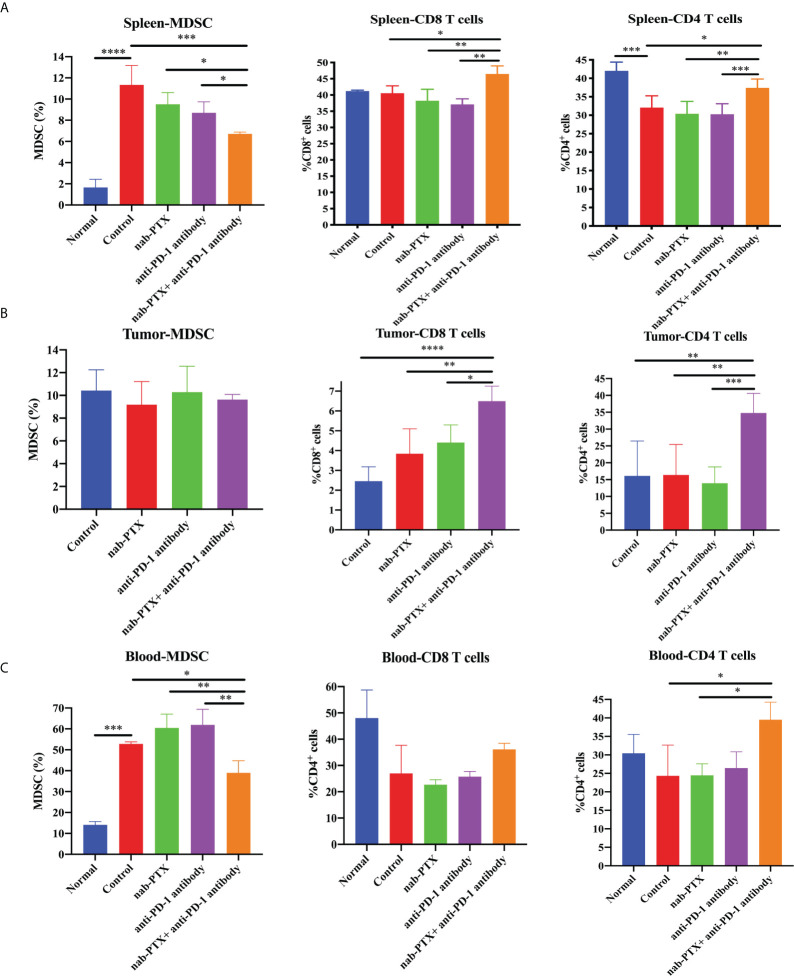
The combination of nab-PTX and anti-PD-1 antibody regulates immune infiltration. The frequency of MDSCs, CD8^+^ T cells, and CD4^+^ T cells in the **(A)** spleen (*n* = 3, biological duplicates), **(B)** tumor (*n* = 5, biological duplicates), and **(C)** blood (*n* = 3, biological duplicates) using one-way ANOVA followed by Tukey’s multiple comparisons. *p< 0.05, **p< 0.01, ***p< 0.001, ****p< 0.0001.

To assess the influence of combined agents on immune function in the process of tumor development, we next examined tumor infiltration in murine peripheral blood, spleens, and tumors using flow cytometry. We found that tumor-bearing mice had markedly increased myeloid-derived suppressor cells (MDSCs) in the spleen and blood compared with non-tumor-bearing mice, and the combination treatment significantly decreased the infiltration of MDSCs compared with any treatment group (**Figures 2A, C**, **3A, C**, *p*< 0.05). As for tumors, there was no obvious reduction in MDSCs (**Figures 2B**, **3B**). In the spleen tissues, we found significantly higher percentages of CD4^+^ and CD8^+^ T cells in mice treated with combination treatment compared with those that received monotherapy or untreated controls, while tumor-bearing mice had decreased CD4^+^ T cells (**Figures 3A**, **S2A, B**, *p*< 0.05). Furthermore, the percentages of CD4^+^ and CD8^+^ T cells were induced by the combination treatment in tumor tissues (**Figures 3B**, **S2C, D**, *p*< 0.05), and the combined therapy increased the proportion of CD4^+^ T cells in the peripheral blood of mice, without affecting CD8^+^ T cells (**Figures 3C**, **S2E, F**, *p*< 0.05). The results showed that the synergistic effects against LLC murine tumor model contributed to the activation of adaptive immunity.

### Combination treatment modulates cytokines in the tumor microenvironment

**Figure 4:**
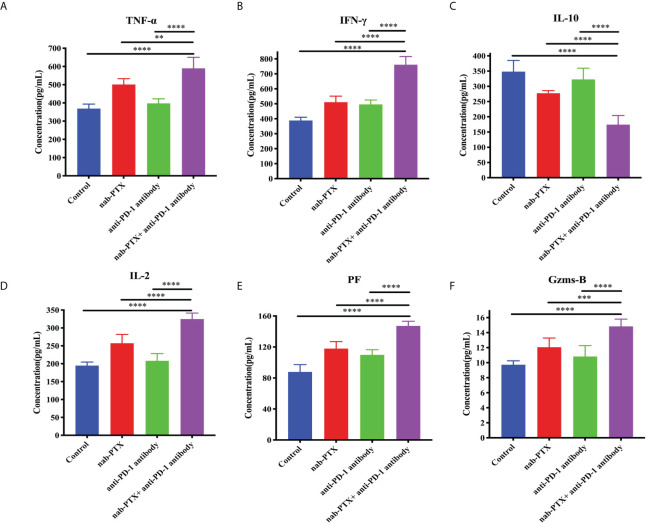
Concentrations of **(A)** TNF-α, **(B)** IFN-γ, **(C)** IL-10, **(D)** IL-2, **(E)** PF, and **(F)** Gzms-B in the tumor microenvironment with the administration of nab-PTX and anti-PD-1 antibody (*n* = 3, biological duplicates). TNF-α, tumor necrosis factor α; IFN-γ, interferon-gamma; IL-10, interleukin 10; IL-2, interleukin 2; PF, perforin; Gzms-B, *granzymes B.* The data were from three independent biological replicates with one-way ANOVA followed by Tukey’s multiple comparisons. **p< 0.01, ***p< 0.001, ****p< 0.0001.

Furthermore, cytokine secretion is also a typical indication of antitumor immune responses. Thus, we subsequently detected the relative changes of TNF-α, IFN-γ, IL-10, IL-2, PF, and Gzms-B concentrations by ELISA kits. We found that TNF-α, IFN-γ, and IL-2, which play vital roles in immunity against tumor growth, were relatively upregulated with these treatments (**Figures 4A, B, D**, *p*< 0.05). The change of IL-10 concentration, a cytokine involved in immunosuppression, was relatively decreased by the administration of nab-PTX and anti-PD-1 antibody (**Figure 4C**, *p*< 0.05). In addition, the combination also induced the secretion of PF and Gzms-B (**Figures 4E, F**, *p*< 0.05). The above experimental results illustrated that the combination of nab-PTX and anti-PD-1 antibody regulated the immune microenvironment, including several cytokines, and thereby may activate and enhance antitumor immune responses.

### Tumor tissues from mice treated with combined agents have a differential gene signature

**Figure 5:**
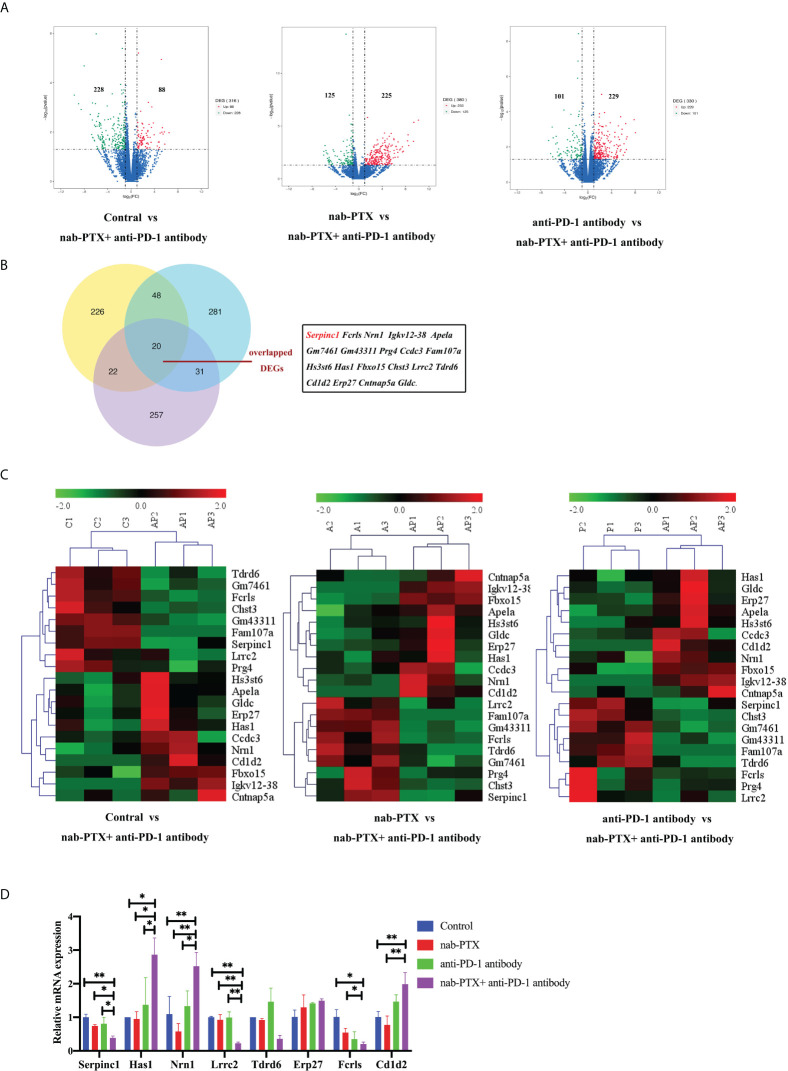
Transcriptomic analysis of mRNA in tumor tissues treated with nab-PTX and anti-PD-1 antibody. **(A)** Volcano plots of differentially expressed genes. Red dots represent upregulated genes, green dots represent downregulated genes, and blue dots represent genes with no significant changes in expression. **(B)** Venn diagram shows the 20 co-expressed DEGs among the three comparisons. **(C)** Gene expression patterns of the overlapped 20 DEGs between every two comparison groups using clustering heatmaps. Red in the heatmap indicates high expression, and green indicates low expression. **(D)** Validation of partial DEGs by qRT-PCR (*n* = 3, biological duplicates), statistically analyzed with two-way ANOVA followed by Tukey’s multiple comparisons. *p< 0.05, **p< 0.01.

To further illustrate the underlying mechanisms of the synergistic antitumor efficacy, we performed a transcriptomic analysis of murine tumor tissues. RNA was extracted from the tumor tissues and prepared for library construction. Through sequencing and differential gene expression analysis, we obtained differentially expressed genes from the pairwise comparisons between the different groups separately. Volcano plots showed the numbers of upregulated and downregulated DEGs caused by combined agents, with three datasets in pairwise comparison between groups (**Figure 5A**). By taking the intersection of these three DEG datasets, 20 co-expressed DEGs were selected (**Figure 5B**), which were *Serpinc1*, *Fcrls*, *Nrn1*, *Igkv12-38*, *Apela*, *Gm7461*, *Gm43311*, *Prg4*, *Ccdc3*, *Fam107a*, *Hs3st6*, *Has1*, *Fbxo15*, *Chst3*, *Lrrc2*, *Tdrd6*, *Cd1d2*, *Erp27*, *Cntnap5a*, and *Gldc*. In addition, the heatmap visualization tool showed detailed gene expression patterns underlying individual functions of these 20 overlapped DEGs, indicating similar clustering (**Figure 5C**). The mRNA expression levels of 10 DEGs (*Serpinc1*, *Has1*, *Nrn1*, *Lrrc2*, *Tdrd6*, *Erp27*, *Fcrls*, *Fam107a*, *Chst3*, *Cd1d2*) in tumor tissues by qRT-PCR were consistent with our transcriptomic analysis, while the expression levels of seven DEGs (*Apela*, *Prg4*, *Gldc*, *Cntnap5a*, *Fbxo15*, *Ccdc3*, *Hs3st6*) were opposite with transcriptomic analysis (**Figures 5D**, **S3**, data not shown partially). Moreover, the other three genes (*Igkv12-38*, *Gm7461*, *Gm43311*) have no primer information for qPCR.

**Figure 6:**
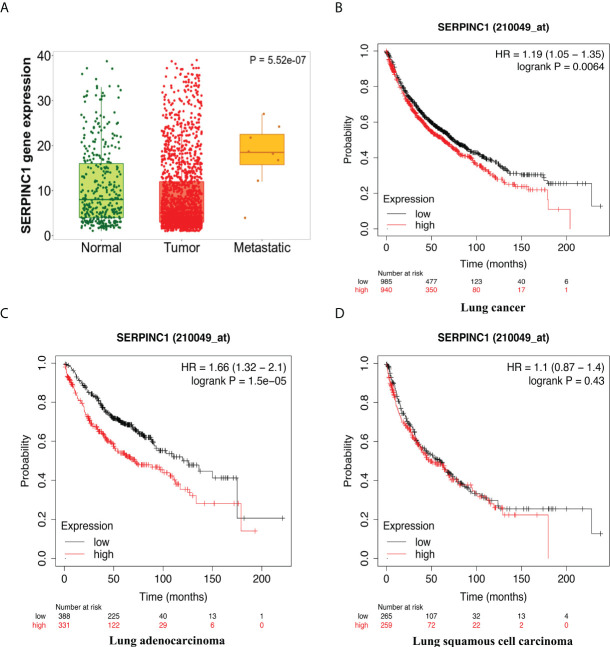
The clinical outcomes of high *Serpinc1* gene expression in public databases. **(A)** Comparisons of *Serpinc1* gene expression in normal (*n* = 391), tumor (*n* = 1,865), and metastatic tissues (*n* = 8) in lung cancer patients using one-way ANOVA followed by Dunnett’s multiple comparisons. Kaplan–Meier survival analysis of OS according to *Serpinc1* expression level in **(B)** lung cancer patients (*n* = 2,437), **(C)** lung adenocarcinoma patients (*n* = 719), and **(D)** lung squamous cell carcinoma patients (*n* = 524).

Furthermore, we chose to study one gene in depth in order to understand the functions of these genes in the antitumor process. Among the co-expressed genes, *Serpinc1* was selected as it was a positive hit in our analyses. *Serpinc1* encodes antithrombin III, whose expression level in tumors was significantly downregulated by combination treatment *in vivo* (**Figure 5D**). Moreover, from the gene chip data of the GEO database, we observed a higher *Serpinc1* expression level in metastatic patients with lung cancer (**Figure 6A**), suggesting a positive association between *Serpinc1* gene expression and tumor metastasis. Kaplan–Meier’s plots revealed a significant inverse correlation of *Serpinc1* gene expression and overall survival in lung cancer patients (**Figure 6B**), especially in patients with lung adenocarcinoma (**Figure 6C**), but there was no significant inverse correlation in patients with lung squamous cell carcinoma (**Figure 6D**). These data raised the possibility that the *Serpinc1* gene may play a role in tumor metastasis and poor survival in lung cancer patients.

### GO functional enrichment analysis reveals that the *Serpinc1* gene correlates with tumor progression

**Figure 7:**
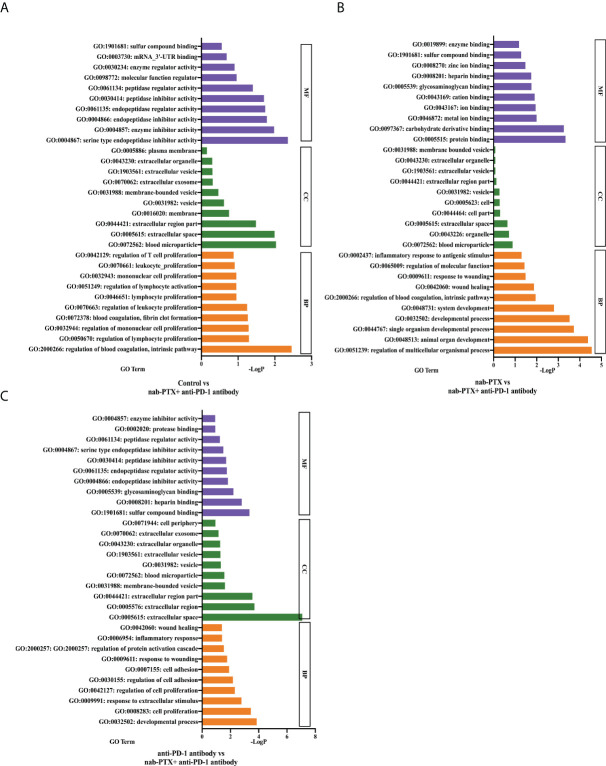
GO analysis of the functional classification of the target gene. The top 10 enriched GO terms of the *Serpinc1* gene in molecular function (MF), cellular component (CC), and biological process (BP) in the comparison between **(A)** the control and the combination group, **(B)** nab-PTX and the combination group, and **(C)** anti-PD-1 antibody and the combination group.

To determine the significant and accurate functions of the *Serpinc1* gene, GO enrichment analysis was performed. In the datasets of the pairwise comparison between the control and combination treatment (**Figure 7A**), *Serpinc1* related to biological processes (BP) was primarily enriched in the regulation of blood coagulation, intrinsic pathway (*p*< 0.01), whereas the target gene related to cellular components (CC) was enriched in the function of blood microparticle (*p*< 0.01). In addition, molecular function (MF) analysis showed that *Serpinc1* was involved in serine-type endopeptidase inhibitor activity (*p*< 0.01). nab-PTX and combination treatment comparison datasets indicated that the gene related to BP was enriched in the developmental process (*p*< 0.0001), response to wounding (*p*< 0.05), and regulation of molecular function (*p*< 0.05). It was enriched in blood microparticle in terms of cellular components, whereas it was also enriched in protein binding in terms of molecular function (**Figure 7B**, *p*< 0.001). Furthermore, the enrichment analysis of the datasets between anti-PD-1 antibody and combined drugs showed that *Serpinc1* related to BP was involved in cell proliferation (*p*< 0.001), regulation of cell adhesion (*p*< 0.01), and response to wounding (*p*< 0.05). The enriched function of CC included extracellular space (*p*< 0.0001), while the enriched function of MF was sulfur compound binding (**Figure 7C**, *p*< 0.001). Through the functional enrichment analysis, we determined several critical tumor-related functions of the *Serpinc1* gene, such as cell adhesion, proliferation, and response to wound healing, worthy of being further investigated.

### *Serpinc1* may play a vital role in the antitumor efficacy *in vitro*

**Figure 8:**
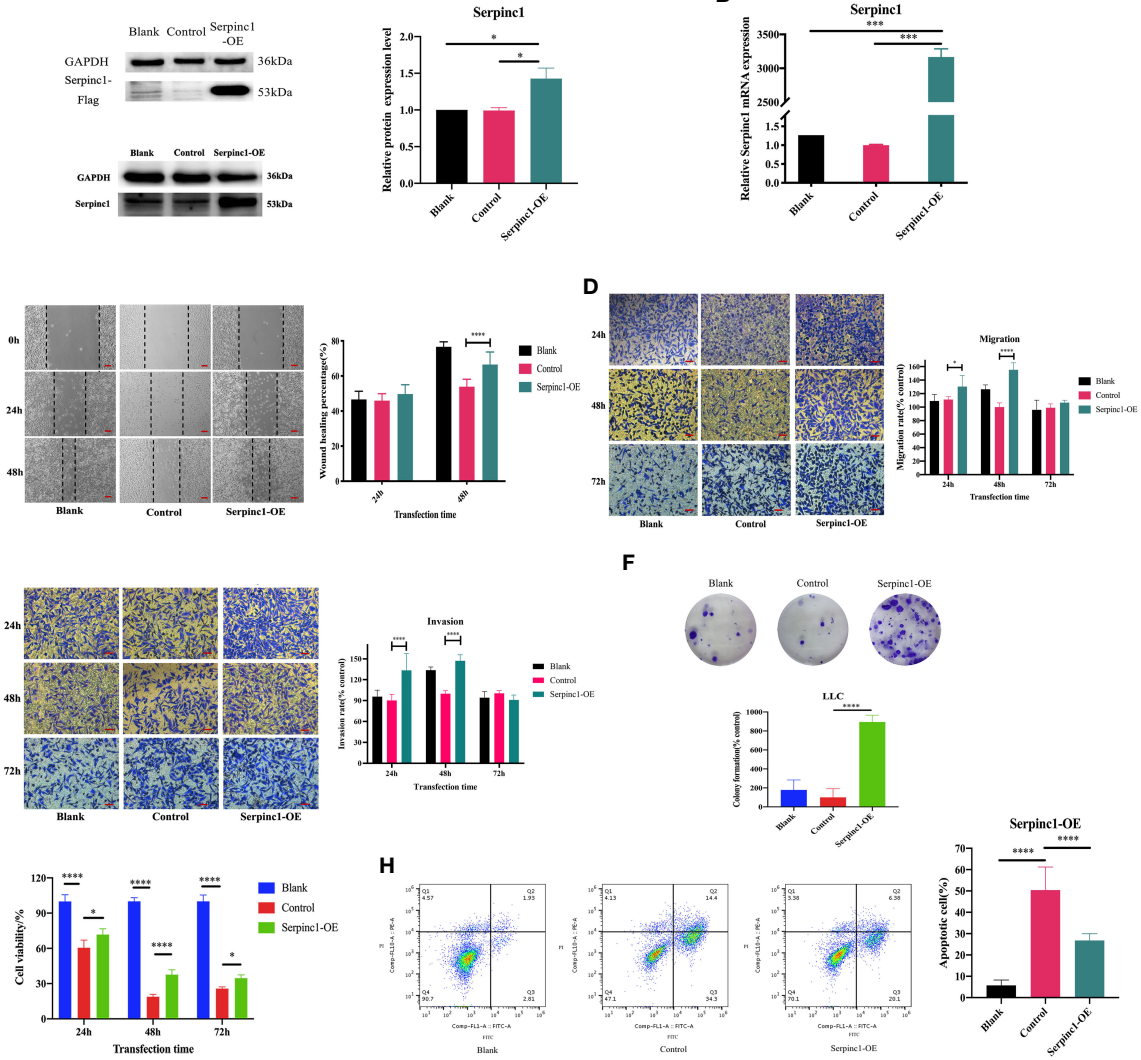
Effects of *Serpinc1* gene on cell migration, invasion, proliferation, and apoptosis. The transfection of *Serpinc1* was determined using **(A)** Western blot analysis and **(B)** qRT-PCR by one-way ANOVA (*n* = 3). **(C)** Wound healing assay was measured and analyzed using two-way ANOVA (*n* = 3, magnification ×200). The rates of **(D)** migration and **(E)** invasion were determined with transfection for 24, 48, and 72 h with two-way ANOVA (*n* = 3, magnification ×400). **(F)** Colony formation assay (*n* = 3) and **(G)** CCK8 assay (*n* = 5) of *Serpinc1* overexpressing LLC cells were used to determine cell viability with ANOVA. **(H)** Apoptotic cells post-transfection were detected on flow cytometry using one-way ANOVA (*n* = 6). *p< 0.05, ***p< 0.001, ****p< 0.0001. *Serpinc1*-OE, *Serpinc1* overexpression.

Based on this positive correlation between *Serpinc1* gene expression and metastasis shown in public databases and the above enriched functions, we hypothesized that the *Serpinc1* gene performs important roles in the regulation of cancer progression *in vitro*, including proliferation, apoptosis, and metastasis. We next focused on the identified pathway analyses. Both cell migration and invasion were directly related to tumor metastasis. We sought to functionally validate the role of *Serpinc1* in cancer progression with LLC cells. We firstly constructed LLC cells overexpressing the *Serpinc1* gene and confirmed successful transfection by Western blotting and qRT-PCR (**Figures 8A, B**, *p*< 0.05). Subsequently, we performed wound healing, migration, and invasion assays using *Serpinc1* overexpressing LLC cells. Cells with *Serpinc1* overexpression plasmid resulted in a faster closure of scratch wounds at 48 h than cells transfected with the control vector (**Figure 8C**, *p*< 0.05). Consistently, cell migration and invasion assays showed that compared with the control vector group, translocated cells were significantly increased in the *Serpinc1* overexpressing group, i.e., *Serpinc1* overexpressing cells displayed markedly induced ability of migration and invasion 24 and 48 h post-transfection (**Figures 8D, E**, *p*< 0.05).

Additionally, we also investigated the effects of *Serpinc1* overexpression on cell proliferation and apoptosis. We observed that *Serpinc1* gene overexpression promoted long-term proliferation (**Figure 8F**, *p*< 0.0001) and increased cell viability of LLC cells compared with the control vector group; however, the cell survival rates of both groups were lower than the non-transfected blank group (**Figure 8G**, *p*< 0.05). Apoptotic cells were also increased in the control vector group than in the non-transfected blank group (**Figure 8H**, *p*< 0.0001). The lower cell viability and more apoptotic cells with transfection in **Figures 8F–H** may be all due to the inevitable toxicity of transfection reagents. Furthermore, *Serpinc1* overexpression markedly reduced the apoptotic cells compared with the control vector group (**Figure 8H**, *p*< 0.001). Taken together, these results demonstrated that the *Serpinc1* gene may affect cell migration, invasion, proliferation, and apoptosis of LLC cells, possibly associated with the antitumor efficacy of the combination treatment.

### *Serpinc1* overexpression affects metastasis, cell cycle, and apoptosis-associated genes and proteins through Pi3K/AKT phosphorylation

**Figure 9:**
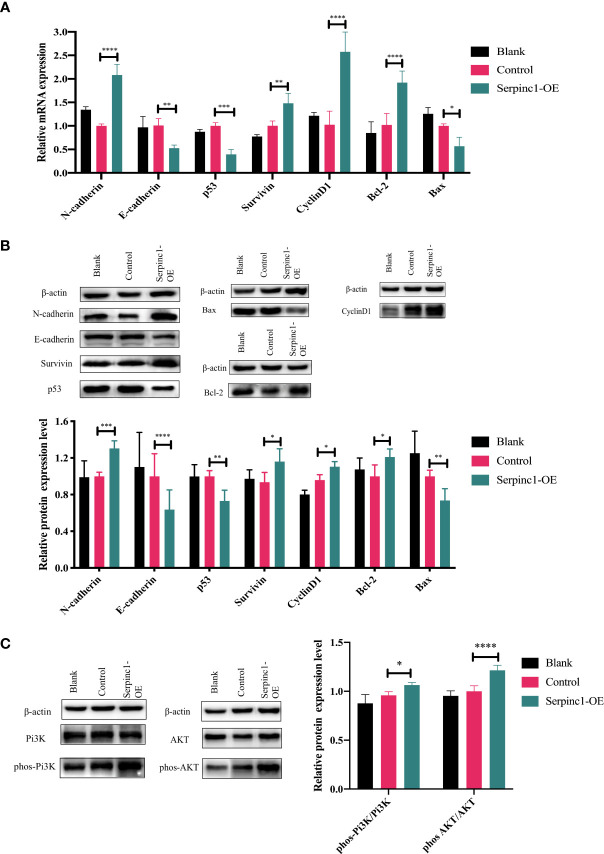
Effects of *Serpinc1* gene overexpression on the expression of genes and proteins involved in cellular migration, invasion, cycle, and apoptosis. **(A)** N-cadherin, E-cadherin, p53, Survivin, CyclinD1, Bcl-2, and Bax mRNA in non-transfected blank, empty vector control, and *Serpinc1* overexpressing cells (*n* = 3). **(B)** Expression levels of N-cadherin, E-cadherin, p53, Survivin, CyclinD1, Bcl-2, and Bax proteins were determined by Western blotting (*n* = 3). **(C)** The phosphorylation of Pi3K and AKT was determined by Western blot analysis (*n* = 3). *p*-values were analyzed by two-way ANOVA with Tukey’s multiple comparisons; comparison between the control vector and *Serpinc1* overexpression. *p< 0.05, **p< 0.01, ***p< 0.001, ****p< 0.0001. Bcl-2, B-cell lymphoma 2; Bax, Bcl-2-associated X protein; AKT, protein kinase B; Pi3K, phosphatidylinositol 3-kinase.

Metastasis (N-cadherin and E-cadherin), cell cycle (CyclinD1 and p53), and apoptosis (Bcl-2, Bax, and Survivin)-associated gene and protein expression levels were determined to further investigate the molecular effects of the *Serpinc1* gene. The relative expression levels of N-cadherin, Survivin, CyclinD1, and Bcl-2 mRNA were significantly upregulated in *Serpinc1* overexpressing cells compared with those in the control vector group, while those of E-cadherin, p53, and Bax mRNA were downregulated (**Figure 9A**, *p*< 0.05). Similar alternations in the expression levels of these proteins were also observed (**Figure 9B**, *p*< 0.05). Moreover, the phosphorylation levels of Pi3K and AKT were induced in cells with *Serpinc1* overexpression (**Figure 9C**, *p*< 0.05), suggesting that *Serpinc1* overexpression activated the Pi3K/AKT pathway. All these data indicated that overexpression of the *Serpinc1* gene in LLC cells may exert functions in cell migration, invasion, proliferation, and apoptosis *via* the Pi3K/AKT pathway, regulating metastasis, cell cycle, and apoptosis-associated factors.

## Discussion

In recent years, the proportion of combination treatment has increased. Previous studies, especially clinical trials, have shown that the combination of chemotherapy and immune checkpoint inhibitors improves the outcomes of patients with several cancers (18–21), with few mechanistic studies. To address the limitation of unclear mechanisms of nab-PTX and anti-PD-1 antibody combination, we made several attempts in our study by establishing *in-vivo* and *in-vitro* models. Here, we provided multiple lines of evidence demonstrating the synergies of nab-PTX combined with the anti-PD-1 antibody. We firstly demonstrated that the synergistic effects of nab-PTX and anti-PD-1 antibody reduced tumor volume growth by up to 60% in an *in-vivo* model of lung cancer.

It has been reported that the efficacy of chemotherapeutic drugs does not reflect only in direct cytotoxic effects but also in increasing immunogenicity of malignant cells, or inhibiting immunosuppressive circuitries (22–24), which might lead to enhanced efficacy of the combination treatment. Chemotherapeutic drugs can stimulate tumor-infiltrating lymphocytes and inhibit immunosuppressive cells (24, 25), while PD-1/PD-L1 interaction promotes differentiation of CD4^+^ T cells into FoxP3^+^ Tregs (26, 27), inhibits tumor-specific T-cell activities, and induces T-cell apoptosis (28), further suppressing the immune system and resulting in peripheral immune tolerance in cancer patients. Consistent with previous findings, we discovered that the combination significantly increased the infiltration of CD4^+^ T cells and CD8^+^ T cells in the TME. The percentages of MDSCs in the spleen and blood confirmed that mice had stronger immunosuppressive effects when they were implanted tumors, and the combination of nab-PTX and anti-PD-1 antibody may inhibit immunosuppression and thereby slow tumor growth. During antigen presentation, nab-PTX plus anti-PD-1 antibody also led to changes in cytokines in the tumor microenvironment, and this trend was in line with relevant reports (29). In addition, it is usually believed that CD8^+^ T cells induce tumor cell death through the following two main methods: i) perforin–granzyme and ii) Fas–FasL (30, 31). The secretion of granzymes and perforin reflects the activation of CD8^+^ T cells. Thus, the increasing concentrations of Gzms-B and PF indicated that nab-PTX combined with anti-PD-1 antibody promoted CD8^+^ T-cell activation. All results of cytokines provided strong evidence to support that nab-PTX synergized potently with anti-PD-1 antibody *in vivo* through the immune system.

More than that, we also attempted to explore the possible molecular mechanisms. In the exploration of underlying mechanisms, we determined 20 co-expressed DEGs through transcriptomic analysis of post-treatment tumor tissues. By qPCR validation, gene expression comparison from the gene chip data (**Figure 6A**), and Kaplan–Meier curves (**Figures 6B–D**), we identified a hit gene, *Serpinc1*, to delve into the biological role. Therefore, GO enrichment analysis was used to predict its function, and it was found that *Serpinc1* was mainly enriched in the regulation of blood coagulation, cell proliferation, adhesion, response to wounding, and response to stimulus. Additionally, it was reported that *Serpinc1* may be related to the development of colorectal cancer (32), breast cancer (33), head and neck cancer (33), ovarian cancer (34), nasopharyngeal cancer (35), liver cancer (36), and endometrial cancer (37), involving the proliferation, migration, and invasion of tumor cells. In a study about lung adenocarcinoma patients (38), eight TME-related prognostic genes were identified by LASSO regression and random forest algorithm, including the *Serpinc1* gene. Though these studies have not stated clearly the relationship between the *Serpinc1* gene and lung cancer, they provide a theoretical basis that it is feasible to assume that *Serpinc1* may be a critical gene for the progression, metastasis, and prognosis of lung cancer.

In our study, *Serpinc1* expression was regulated by the combination treatment *in vivo* and involved in cell proliferation, apoptosis, migration, and invasion of LLC cells. *Serpinc1* gene overexpression modulated the expression of N-cadherin, E-cadherin, Survivin, p53, CyclinD1, Bcl-2, and Bax, elaborating that the *Serpinc1* gene may contribute to the pathogenesis of lung cancer. Furthermore, the Pi3K/AKT pathway has been reported to participate in the pathogenesis of numerous cancer types (39, 40), crucial to tumor cell growth, survival, death, and epithelial-to-mesenchymal transition (41–43). In recent years, the link between the Pi3K/AKT pathway and coagulation has been reported in several studies (44, 45). Therefore, *Serpinc1*, a most important serine protease inhibitor that regulates the blood coagulation cascade, is possibly related to this signaling pathway. Actually, we observed that *Serpinc1* overexpression activated the phosphorylation levels of Pi3K/AKT (**Figure 9C**), thereby illustrating that the possible mechanism of the *Serpinc1* gene involved in cancer progression may be due to signal transduction of the Pi3K/AKT pathway. Thus, *Serpinc1* may be a meaningful indicator for the therapeutic efficacy of this combination. Furthermore, this target gene will be investigated in greater depth by verifying on humanized cells, other lung cancer cell lines, and metastatic tumor models, taking dosage modulation into consideration as well. The association between *Serpinc1* and the Pi3K/AKT pathway and how the Serpinc1 protein exerts its effects on cancer cells require further investigation. We will attempt to address these issues in future research.

Here, the transcriptomic analysis of mouse LLC lung cancer models was performed to generate a resource of antitumor synergistic genes, which facilitates elucidation of the underlying mechanisms of the combination. We anticipate that further investigation of *Serpinc1* and other now-uncovered genes/pathways will help reveal the synergy of nab-PTX and anti-PD-1 antibody in tumor types other than lung cancer. In conclusion, this study provides a strong support for the synergistic effect of nab-PTX combined with anti-PD-1 antibody in inhibiting tumor growth, enhancing immune responses, and suppressing *Serpinc1* gene expression and emphasizes the role of *Serpinc1* overexpression in cancer development. Therefore, reducing the expression of *Serpinc1* may be considered as an effective approach to treat lung cancer.

## Data availability statement

The datasets presented in this study can be found in online repositories. The names of the repository/repositories and accession number(s) can be found below: https://www.ncbi.nlm.nih.gov/, PRJNA833745.

## Ethics statement

The animal study was reviewed and approved by Institutional Animal Care and Use Committee (IACUC), School of Pharmacy, Fudan University.

## Author contributions

JZ designed the experiments and had full access to all the data in the study. Agents were provided by XG and YZ. Study design: JZ and YW. Drafting of the manuscript and statistical analysis: JZ. Revision of the manuscript: ZT. Supervision: WC. All authors contributed to the article and approved the submitted version.

## Funding

This work was supported by grant from the National Natural Science Foundation of China [Grant No. 8217130423].

## Conflict of interest

The authors declare that the research was conducted in the absence of any commercial or financial relationships that could be construed as a potential conflict of interest.

## Publisher’s note

All claims expressed in this article are solely those of the authors and do not necessarily represent those of their affiliated organizations, or those of the publisher, the editors and the reviewers. Any product that may be evaluated in this article, or claim that may be made by its manufacturer, is not guaranteed or endorsed by the publisher.
