# Corrigendum: Synergistic effects of nab-PTX and anti-PD-1 antibody combination against lung cancer by regulating Pi3K/AKT pathway through the *Serpinc1* gene

**DOI:** 10.3389/fonc.2023.1303608

**Published:** 2023-10-30

**Authors:** Jun Zhang, Zhijia Tang, Xi Guo, Yunxia Wang, Yuhong Zhou, Weimin Cai

**Affiliations:** ^1^ Department of Clinical Pharmacy, School of Pharmacy, Fudan University, Shanghai, China; ^2^ Department of Medical Oncology, Zhongshan Hospital, Fudan University, Shanghai, China

**Keywords:** albumin-bound paclitaxel, combination drug therapy, lung cancer, PD-1, *Serpinc1*

In the published article, there were errors in **Figures 2**, **8** as published. In **Figures 2A, B**, we put the wrong representative plots in the previous **Figure 2**. **Figures 8D, E** showed the rates of migration and invasion with transfection for 24, 48, and 72 h. The third image in the second row of **Figure 8E** is the same as the one in the same position of **Figure 8D**. The image of cell invasion after transfection with Serpinc1-OE 48 h in **Figure 8E** was wrongly used. The corrected **Figures 2**, **8** are shown below.

**Figure 2:**
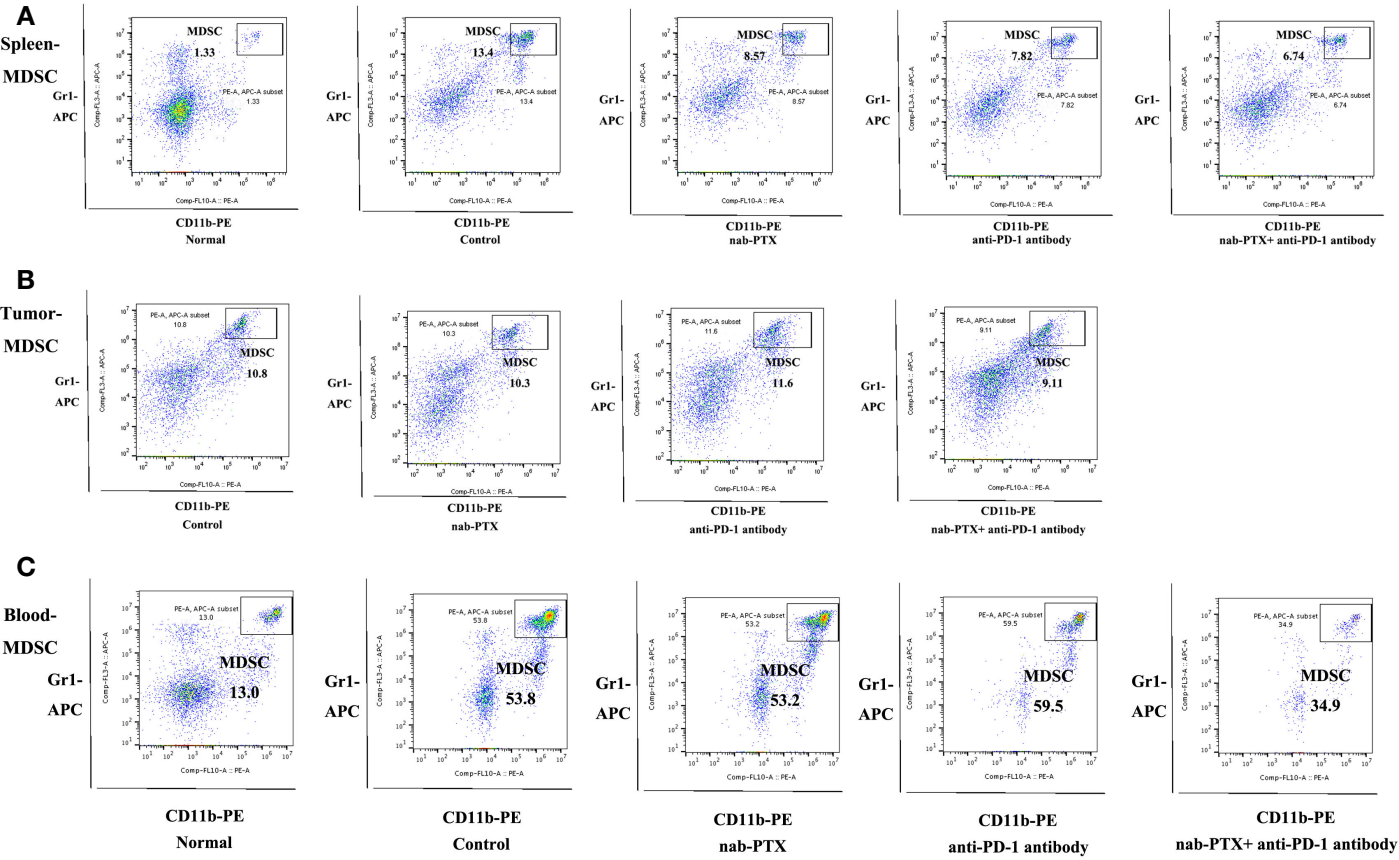
Representative flow cytometric plots of MDSCs in **(A)** spleen (n = 3, biological duplicates), **(B)** tumor (n = 5, biological duplicates), and **(C)** peripheral blood (n = 3, biological duplicates) after treatment with nab-PTX and anti-PD-1 antibody.

**Figure 8:**
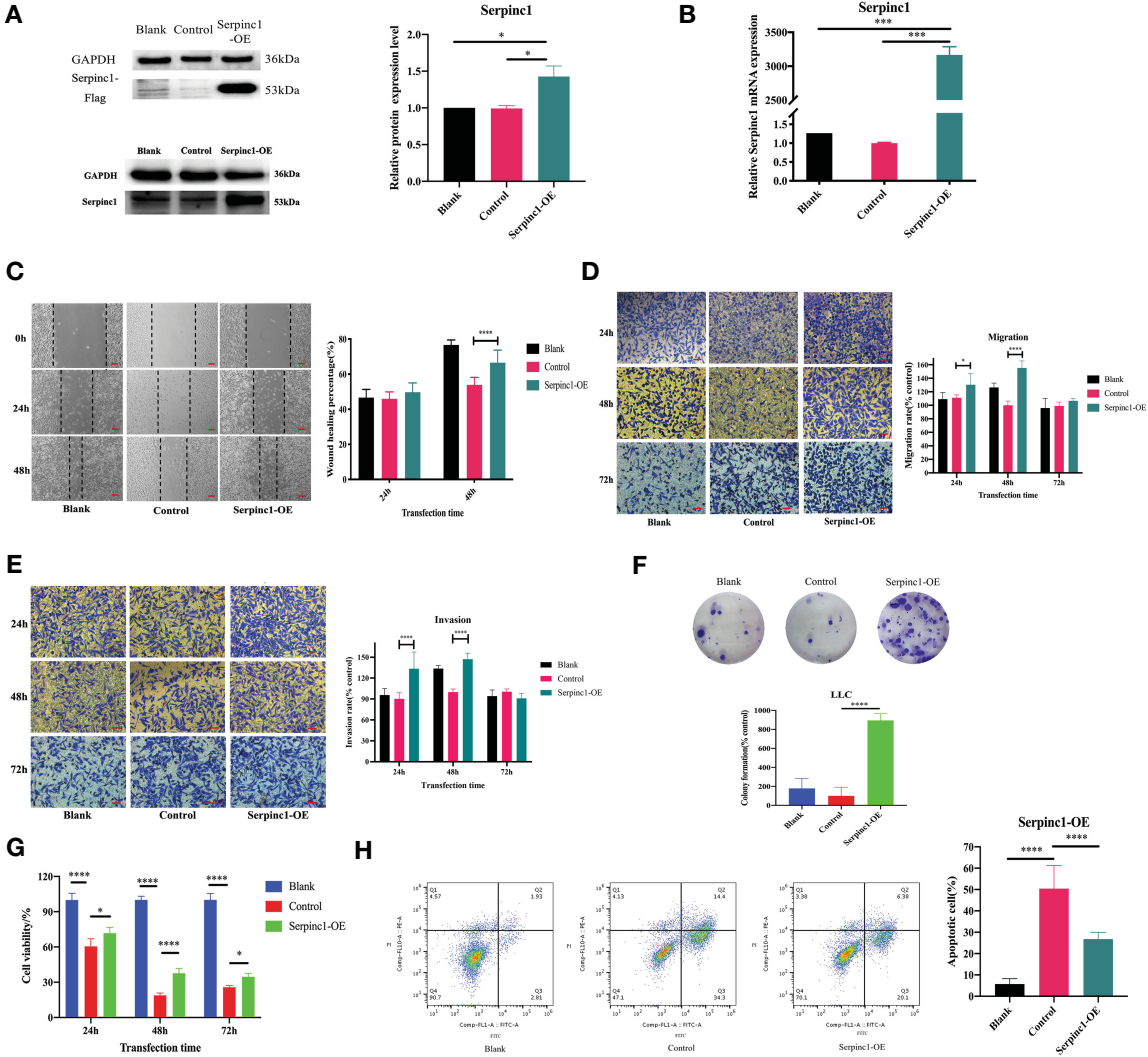
Effects of *Serpinc1* gene on cell migration, invasion, proliferation, and apoptosis. The transfection of *Serpinc1* was determined using **(A)** Western blot analysis and **(B)** qRT-PCR by one-way ANOVA (n = 3). **(C)** Wound healing assay was measured and analyzed using two-way ANOVA (n = 3, magnification ×200). The rates of **(D)** migration and **(E)** invasion were determined with transfection for 24, 48, and 72 h with two-way ANOVA (n = 3, magnification ×400). **(F)** Colony formation assay (n = 3) and **(G)** CCK8 assay (n = 5) of *Serpinc1* overexpressing LLC cells were used to determine cell viability with ANOVA. **(H)** Apoptotic cells post-transfection were detected on flow cytometry using one-way ANOVA (n = 6). *p< 0.05, ***p< 0.001, ****p< 0.0001. Serpinc1-OE, *Serpinc1* overexpression.

The authors apologize for this error and state that this does not change the scientific conclusions of the article in any way. The original article has been updated.

