# Correction: Synergistic effects of nab-PTX and anti-PD-1 antibody combination against lung cancer by regulating Pi3K/AKT pathway through the *Serpinc1* gene

**DOI:** 10.3389/fonc.2026.1815252

**Published:** 2026-03-03

**Authors:** Jun Zhang, Zhijia Tang, Xi Guo, Yunxia Wang, Yuhong Zhou, Weimin Cai

**Affiliations:** 1 Department of Clinical Pharmacy, School of Pharmacy, Fudan University, Shanghai, China; 2 Department of Medical Oncology, Zhongshan Hospital, Fudan University, Shanghai, China

**Keywords:** albumin-bound paclitaxel, combination drug therapy, lung cancer, PD-1, *Serpinc1*

In the published article, there was an error in the **Funding statement**. The funding number was incorrectly written as “8217130423” rather than “82173892”. The correct **Funding statement** appears below.

“This work was supported by grant from the National Natural Science Foundation of China [Grant No. 82173892].”

The original article has been updated.

