# Supplementary material for: Synergistic effects of nab-PTX and anti-PD-1 antibody combination against lung cancer by regulating the Pi3K/AKT pathway through the Serpinc1 gene
Source: Front Oncol. 2022 Aug 3;12:933646. doi: 10.3389/fonc.2022.933646 (PMC9381811; doi:10.3389/fonc.2022.933646)
Supplement: Supplementary file 1 [file DataSheet_1.docx]

Supplementary Material

# Supplementary Figures and Tables

## Supplementary Figures


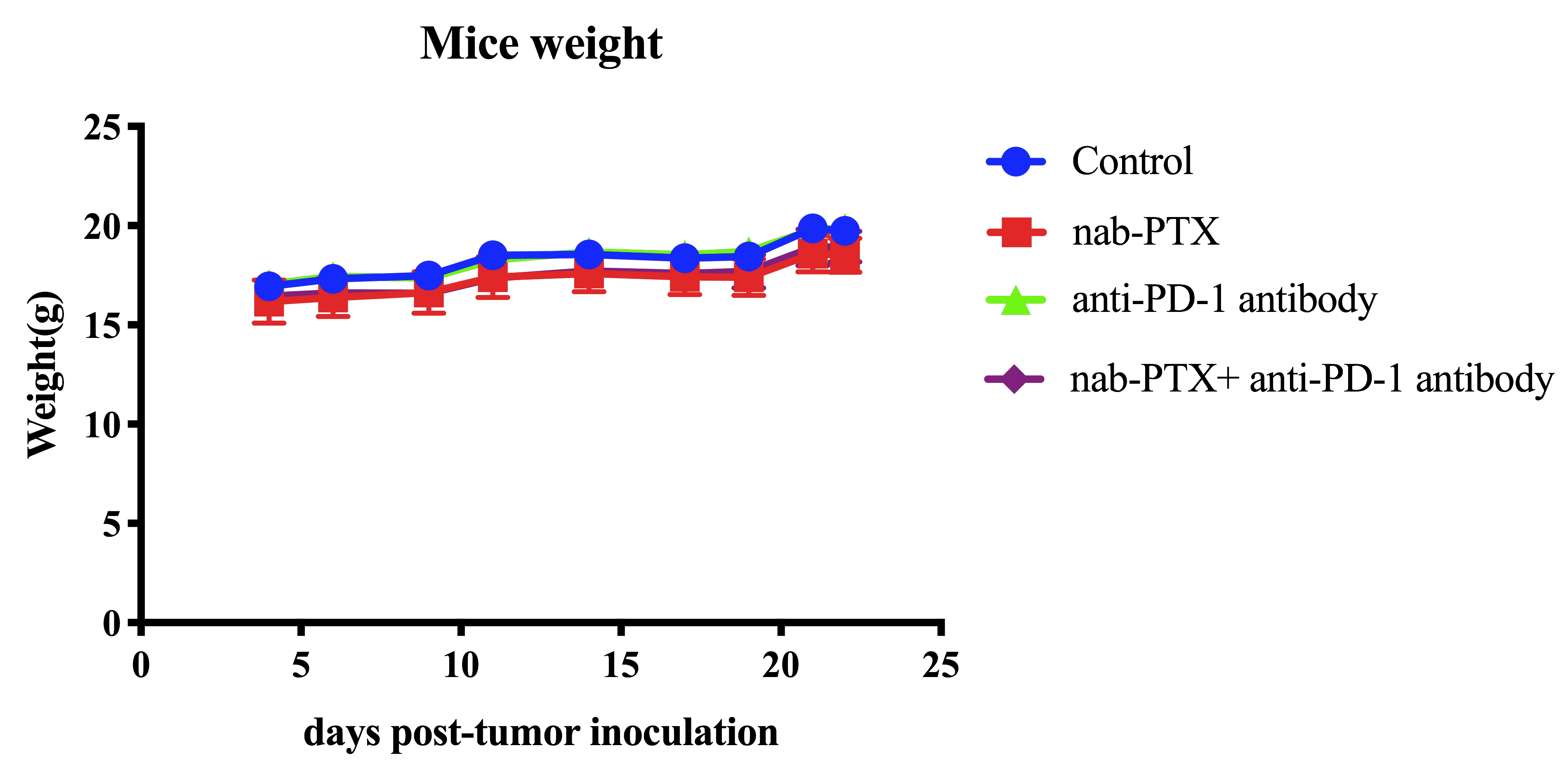


**Supplementary Figure 1.** Changes in body weight of mice with the administration of nab-PTX, anti-PD-1 antibody and combination treatment (n=6, biological duplicates), statistically analyzed with two-way ANOVA followed by Tukey's multiple comparisons.


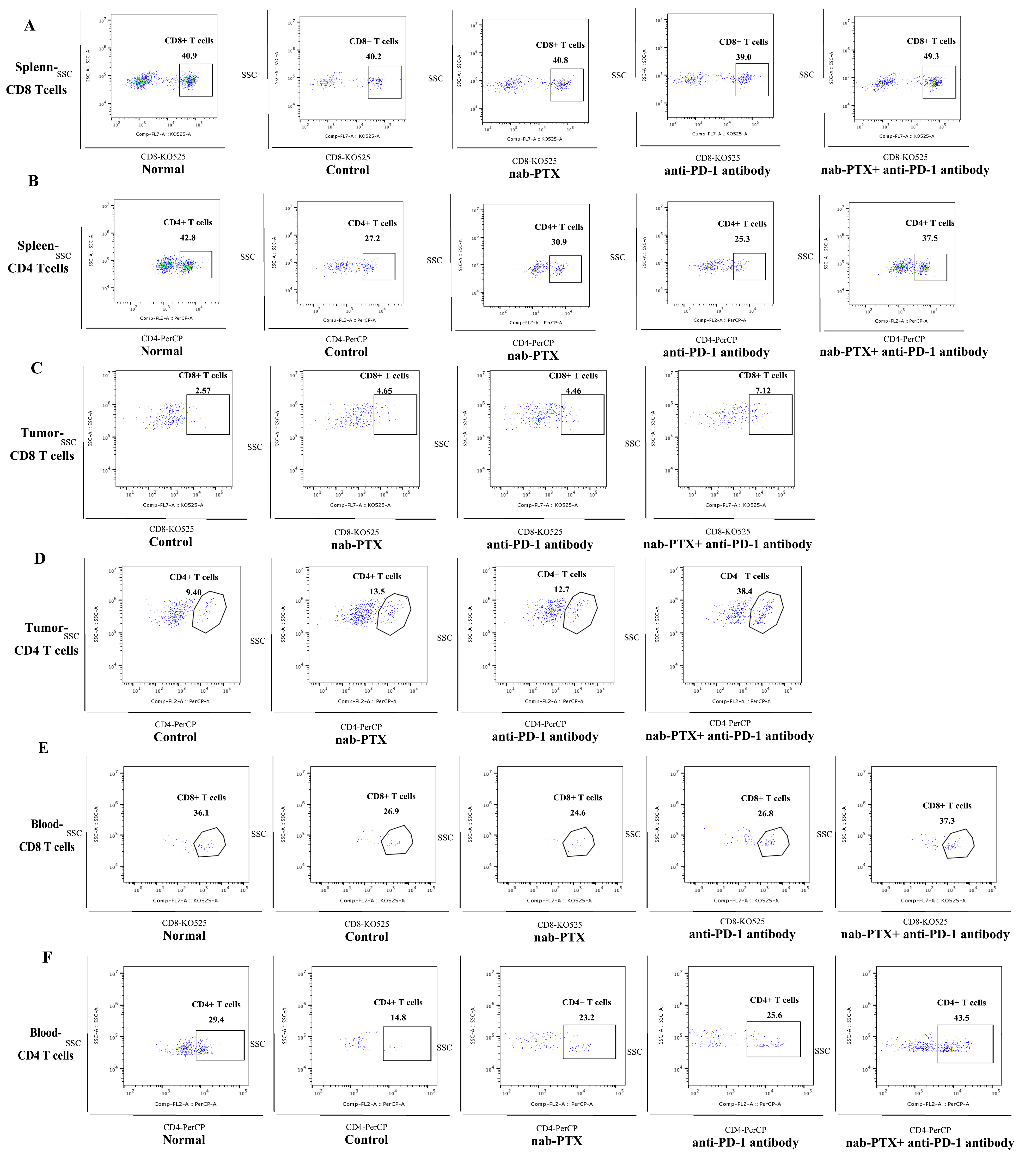


**Supplementary Figure 2.** Representative flow cytometric plots of immune cells. **(A)** CD8+ T cells and **(B)** CD4+ T cells of the spleen with the treatment (n=3, biological duplicates). **(C)** CD8+ T cells and **(D)** CD4+ T cells of the tumor tissues in each group (n=5, biological duplicates). **(E)** CD8+ T cells, and **(F)** CD4+ T cells of blood in each group (n=3, biological duplicates).


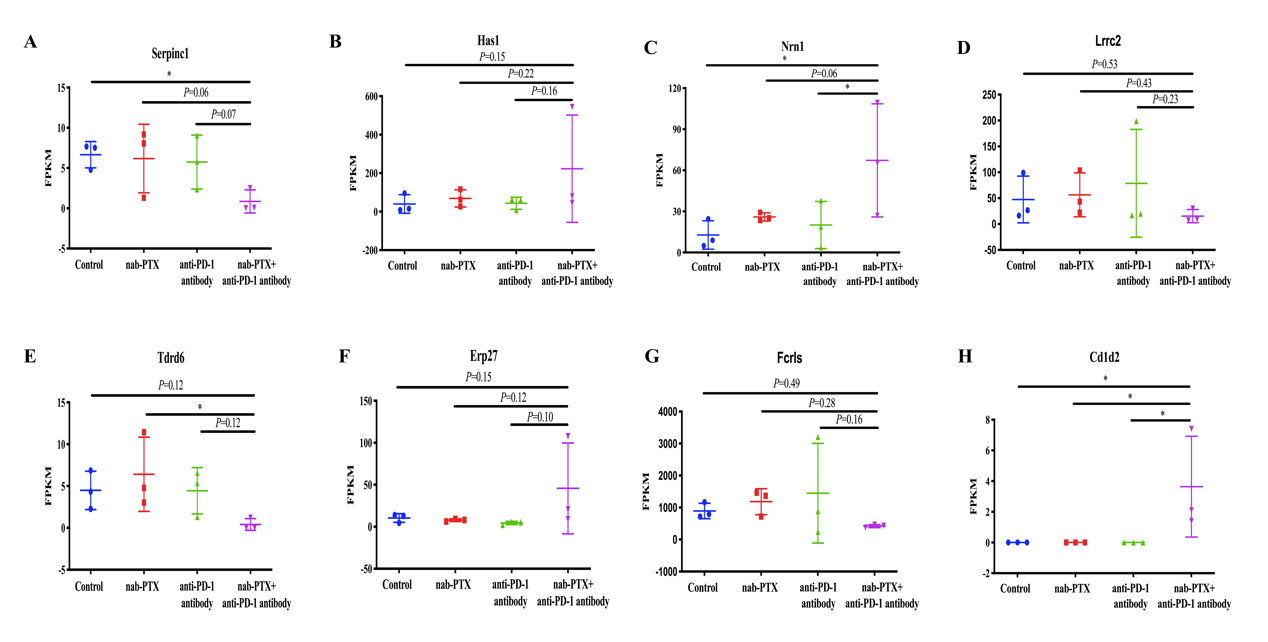


**Supplementary Figure 3.** FPKM levels of the overlapped twenty DEGs in RNA-seq data (the other genes not shown) (n=3, biological duplicates). **(A)** *Serpinc1*, **(B)** *Has1*, **(C)** *Nrn1*, **(D)** *Lrrc2*, **(E)** *Tdrd6*, **(F)** *Erp27*, **(G)** *Fcrls*, **(H)** *Cd1d2*. FPKM, fragments per kilobase of transcript per million mapped reads. The data was analyzed with one-way ANOVA followed by Tukey's multiple comparisons. *p<0.05, **p<0.01, ***p <0.001,****p<0.0001.

## Supplementary Tables

**Supplementary Table 1.** Sequences of the primers used for differential genes.

| Gene | Reverse primer | Forward primer |
| --- | --- | --- |
| GAPDH | TGCCGTGAGTGGAGTCATAC | AGGAGAGTGTTTCCTCGTCCC |
| Nrn1 | CGGTCTTGATGTTCGTCTTGTC | GCGGTGCAAATAGCTTACCTG |
| Serpinc1 | GGATTCACGGGGATGTCTCG | GGCTGCTGGTGAGAGGAAG |
| Prg4 | CCTCTCCACATTGTACTCACTT | CACACCTTCAGGATCCATTACT |
| Apela | CATGGGAAGGGCACTCGAGA | ACCAGTTAACTTTCCCAGGAGAAGA |
| Ccdc3 | GCCACGGCAGGTAGTTGTA | TGGAGACCCTTGAGCGAAG |
| Fam107a | GTGGTTCATAAGCAGCTCACG | CAGACCAGAGTACAGAGAGTGG |
| Has1 | CTGGCCCTGGTCCTACAATC | GGTGGTTACCGTGTCCAAGT |
| Fbxo15 | TGACAGATGAGCCTCTAACAAAC | TCGTGGGACTGAGCACAACTA |
| Chst3 | GTCGGAGACCCTGGATATGATT | GCCTAAAGATTCGAGGCAGATAC |
| Cntnap5a | TCTTCATCTGATTGGTGCGGTGTG | CAGCAGTCATTGGAGGCGTCATAG |
| Gldc | GGACCGTCTTCTCGATGAGC | CTCCTGCCCAGACACGATG |
| Fcrls | ATGGTGTAGCTTGAAGCACTG | CTTCTGGTCTTCGCTCCTGTC |
| Cd1d2 | CCCAGGGTACATTTCACAGCC | GTCCCAGGGCAAGTTGAGTAA |
| Erp27 | CAGCAGCGATGAGTTCCAC | AGATGCCTAATCCTCTCGTTTGT |
| Tdrd6 | GGGATGGCATACTTGGGTTACT | TTCGCTGCTCAAACGCTAC |
| Hs3st6 | CACCACAATCAGCTTGGTATCT | TTCTTCGACAGGTGCTATGATCG |
| Lrrc2 | ACACAAATGGACTCCTCCGTT | AGAAGCACAAAGCATGGCAGA |
